# Reversible cerebral vasoconstriction syndrome after intravenous iron substitution: a case report

**DOI:** 10.1007/s00415-022-11011-3

**Published:** 2022-02-13

**Authors:** Katharina Johanna Müller, Florian Schöberl, Thomas David Fischer, Moritz Luigi Schmidbauer, Dennis Cem Thunstedt, Katharina Eisenhut, Carla Palleis, Andreas Straube, Matthias Klein

**Affiliations:** 1 Department of Neurology, University Hospital Munich, Ludwig-Maximilians Universität München, Campus Grosshadern, Marchioninistr. 15, 81377 Munich, Germany; 2 Institute of Neuroradiology, Ludwig-Maximilians Universität München, Campus Grosshadern, Marchioninistr. 15, 81377 Munich, Germany; 3 Institute of Clinical Neuroimmunology, University Hospital and Biomedical Center, Ludwig-Maximilians-Universität München, Marchioninistr. 15, 81377 Munich, Germany; 4 German Center for Neurodegenerative Diseases (DZNE) Munich, Munich, Germany; 5 Munich Cluster for Systems Neurology (SyNergy), Munich, Germany

Dear Sirs,

Reversible cerebral vasoconstriction syndrome (RCVS) is an important differential diagnosis in patients with acute onset thunderclap headache [1, 2]. Clinical presentation includes sudden onset of most intense headache and may be accompanied by unspecific symptoms such as altered mental state, nausea, photo- or phonosensitivity or dizziness [2]. It predominantly affects women with a female/male ratio of approximately 2:1 and mean age at 42 to 44 years [2]. RCVS can occur in the context of various conditions such as pregnancy, eclampsia, migraine and sexual activity [1]. Furthermore, it has been shown to be associated with multiple vasoactive and immunosuppressive substances such as triptans, selective serotonin reuptake inhibitors, erythrocyte transfusions, amphetamine derivates, epinephrine, tacrolimus etc. [1]. Among others, differential diagnosis include subarachnoid haemorrhage and posterior reversible encephalopathy syndrome (PRES), primary angiitis of the CNS, cerebral venous sinus thrombosis and migraine [1]. Because the differential is broad and potentially life threatening, a rapid evaluation including CT angiography and lumbar puncture is needed. While pathophysiology of RCVS is still not well understood current hypothesis propose that the dysregulation of vascular tone might result from a complex interaction of sympathetic overactivity and endothelial dysfunction [1]. However epidemiologic evidence to proof a causal relationship is still lacking [2].

**Fig. 1:**
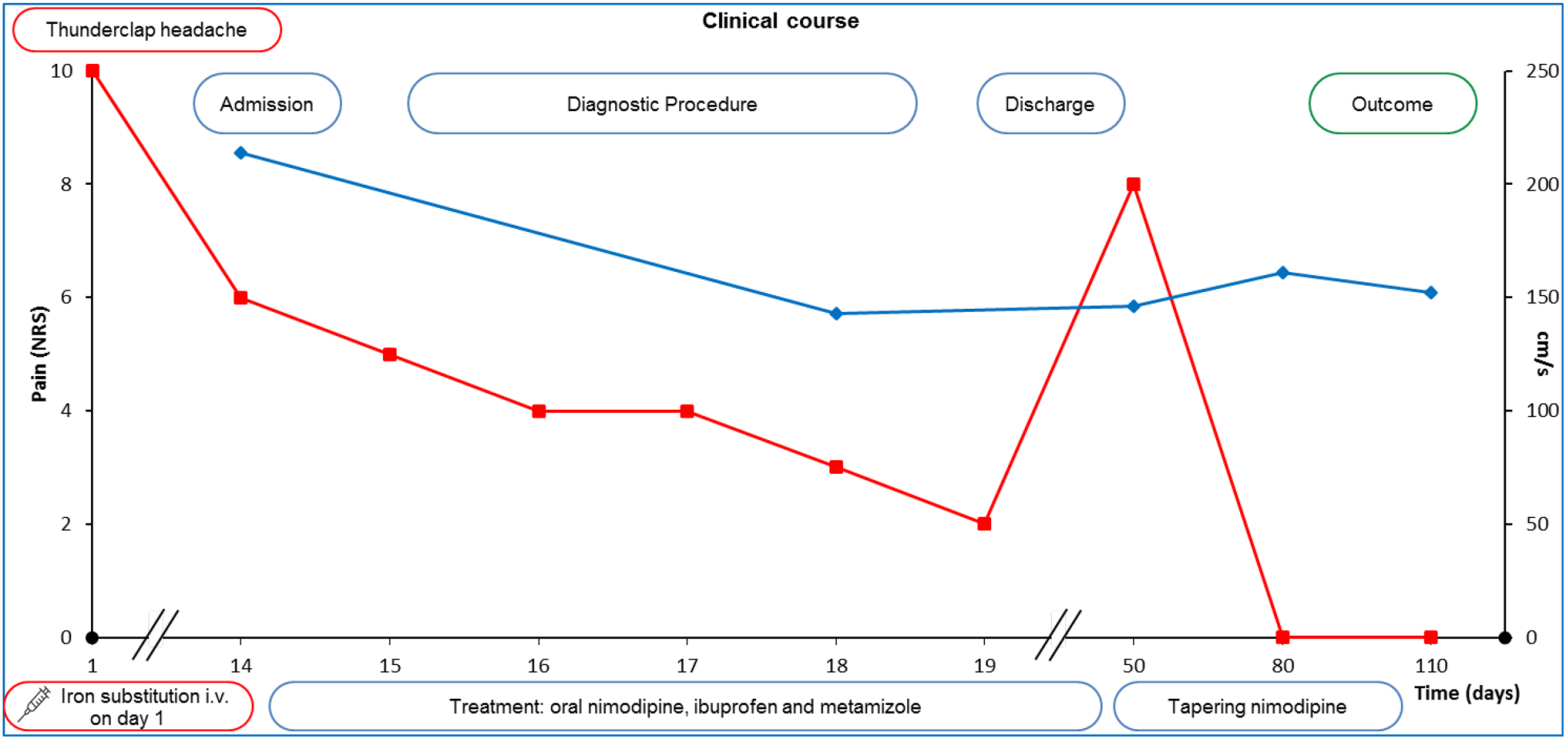
Clinical timeline of a 38-year-old woman diagnosed with RCVS after i.v. iron substitution, pain level (NRS) depicted in red and intracranial PSV of the left MCA in cm/s in blue

A 38-year-old Caucasian female nurse presented to our emergency department with persistent holocephalic headache after having had a first abrupt episode of thunderclap headache two weeks prior to admission. That first episode of thunderclap headache started 20 min after she was substituted with intravenous iron (i.e. 1000 mg Ferric Carboxymaltose) for the first time in order to treat chronic iron deficiency anemia (see Fig. 1). The thunderclap headache lasted 45 min in total with accompanying dizziness and disorientation. The thunderclap headache was followed by 2 weeks of persistent holocephalic headache with a dull-oppressive character and an intensity of 6/10 (numeric rating scale/NRS). Additional symptoms were dizziness, blurry vision and fatigue. She had a past medical history of iron deficiency anemia, menorrhagia and hypothyroidism, her medication included l-thyroxine and previously oral iron [ferrous (II) glycine sulphate complex]. She was an active smoker with 8 pack years and denied the use of alcohol or illicit drugs. There was no past medical history of migraine or other primary headache syndromes. Family history was significant for cerebrovascular disease (ischemic stroke of unknown etiology of her mother at the age of 55).

Examination showed obesity grade I (BMI 31.1), the neurologic examination was unremarkable. Electrocardiogram detected sinus rhythm at a heart rate of 96/min without any further abnormalities. Blood pressure was slightly elevated with 149/82 mmHg. Laboratory test results showed hypochromic, microcytic anemia [hemoglobin 10.1 g/dl, mean corpuscular hemoglobin (MCH) of 78.7gl, mean corpuscular volume (MCV) of 25.2 pg] and an elevated red cell distribution width (RDW) of 19.5%. By CSF analysis (performed on admission two weeks after the first episode) subarachnoid haemorrhage was definitely excluded (cell count: < 1/µl [< 5/µl], no erythrocytes, no xanthochromia, no siderophages; protein level: 36 mg/dl [15–45 mg/dl], glucose: 67 mg/dl [55–110 mg/dl, serum glucose 114 mg/dl]). CSF opening pressure was slightly elevated with 31 cmH_2_O [6-25cmH_2_O].

**Fig. 2:**
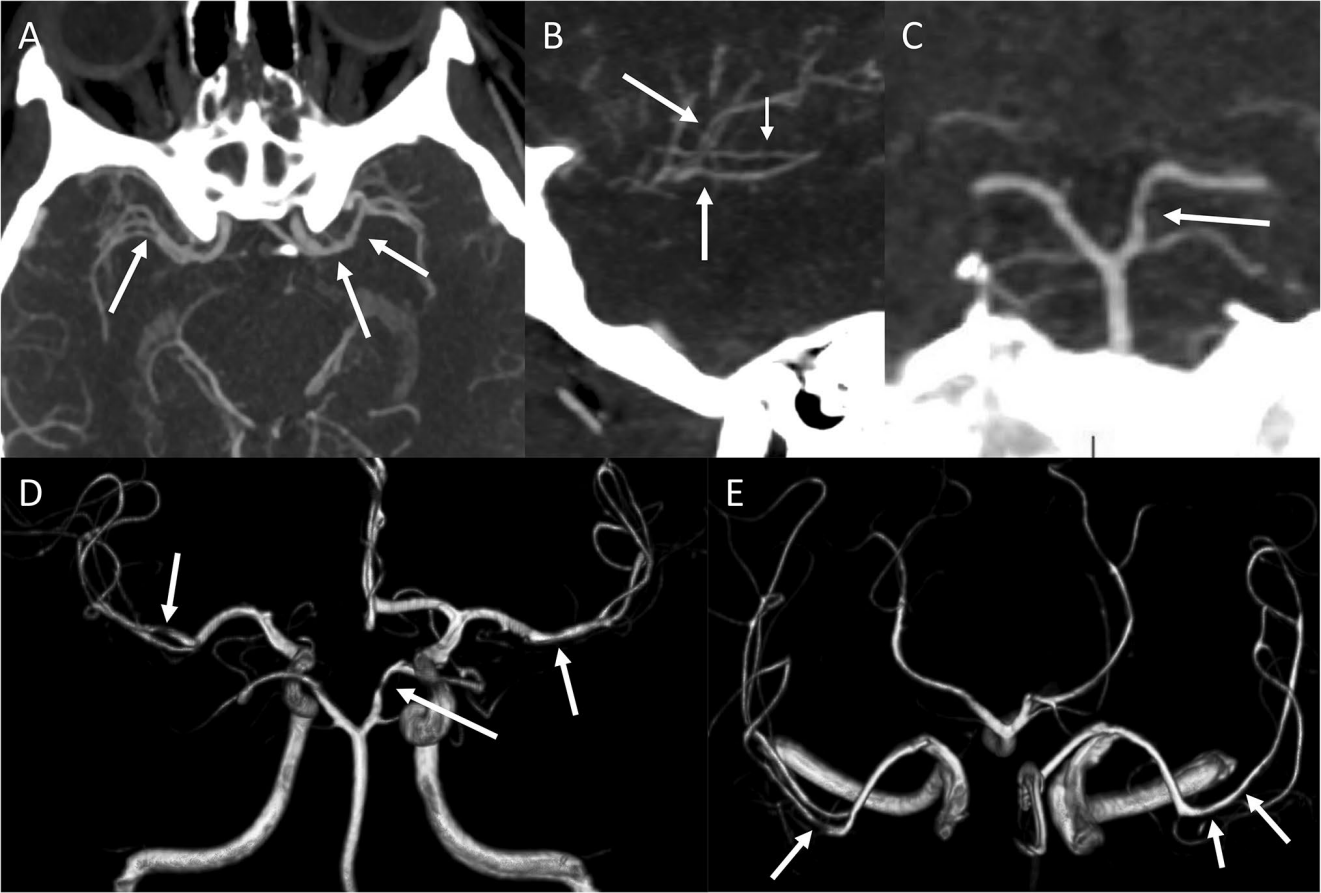
Maximum intensity projections of brain CT angiography at admission demonstrating distinct irregularities of the brain-supplying arteries suspicious of vasospasm. The mainly involved segments were the M1- (**A**) and M2-segments (**B**) of both middle cerebral arteries and the P1-segment of the left posterior cerebral artery (**C**). 3D-time-of-flight-MR angiography on day 5 after admission showing persistent multifocal vasospasms in the M1-segment of both medial cerebral arteries (MCA), the P1-segment of the left posterior cerebral artery (**D**, coronal) and the M2-segment of both MCAs (**E**, axial)

Multimodal CT scan with CT-angiography revealed multifocal segmental cerebral artery vasoconstriction, predominantly affecting both middle cerebral arteries (MCA) and the P1-segment of the posterior cerebral artery (PCA) (Fig. 2 A–C). Transcranial color-coded duplex sonography (TCCD) showed segmental vasospasms with peak systolic velocity (PSV) values up to 214 cm/s measured in the left MCA M1-segment and an elevated Lindegaard-Index of 3.5. Time-of-flight (TOF) magnetic resonance angiography (performed already under treatment with nimodipine on day 5 after admission) revealed segmental decrease of vessel diameter in the MCA on both sides and in the P1-segment of the left PCA (Fig. 2 D–E). Diffusion-weighted imaging (DWI) revealed no signs of acute infarction, susceptibility-weighted imaging (SWI), gradient echo and T2w-fluid attenuated inversion recovery (T2-FLAIR) sequences showed no evidence of subarachnoid hemorrhage (SAH), sulcal siderosis, microbleeds or cerebral venous sinus thrombosis (CVST). In addition, there were no edema suggestive of additional posterior reversible encephalopathy syndrome (PRES) and no signs rising suspect of intracranial hypertension such as optic nerve sheath edema or an empty sella region. Contrast-enhanced T1-weighted black-blood vessel wall imaging revealed no suspect of CNS vasculitis.

Combined, RCVS was diagnosed and treatment with oral nimodipine (3 × 60 mg daily) and additional analgesics (ibuprofen 1200 mg daily and metamizole 3 g daily) was started. Symptoms improved, no further episodes of thunderclap headache occurred at that time and control TCCD showed normalized PSV values after five days (149 cm/s in the left MCA). After discharge, sequential TCCD was performed every four weeks (for 3 months) recording normal PSV values. While tapering oral nimodipine gradually over 10 weeks, the patient experienced one more episode of holocephalic headache with an intensity of 8/10 (NRS), which resolved spontaneously. The symptoms completely resolved within three months and there was no second episode of thunderclap headache (Fig. 1).

To our knowledge, this is the first case describing thunderclap headache and RCVS after intravenous iron substitution in a patient with chronic iron deficiency anemia. The diagnosis of RCVS was established by the combination of the following findings: (i) thunderclap headache, (ii) vasoconstriction on cerebrovascular imaging, and (iii) rule out of subarachnoid haemorrhage by CT and lumbar puncture. A connection to the intravenous iron substitution seems very likely by its timely association in absence of other trigger factors of RCVS.

Iron is a key-element and involved in the regulation of oxygen transport and mitochondrial function acting as a transition-metal ion and as a cofactor in various enzymatic processes (such as ribonucleotide reductase, cytochromes, NADH dehydrogenase) [3]. However it is also a key player regulating oxidative stress by forming and scavenging highly toxic reactive oxygen species (ROS) and reactive nitrogen species (RNS) as well as being a cofactor of lipoxygenases, fatty acid desaturases and superoxide dismutases [3, 4].

Oral and intravenous iron preparations differ regarding the valency of iron ions [ferrous (Fe^2+^) *versus* ferric (Fe^3+^) ions] and their bioavailability, as overall plasma-free and non-transferrin-bound iron is higher after direct intravenous iron administration [5]. Yet, non-transferrin-bound iron has been shown to have dose-dependent toxic effects in chronic renal disease and to be associated with increased oxidative stress, endothelial damage, inflammation and organ failure [6, 7].

Several studies investigated the relationship between iron deficiency anemia, iron substitution and increased oxidative stress: in patients with iron deficiency anemia and chronic renal disease or heart insufficiency intravenous iron administration leads to iron misdistribution and is associated with a production of oxidants resulting in related complications due to iron overload and oxidative stress [7, 8]. Interestingly an iron overload due to erythrocyte transfusions being associated with RCVS is also shown in a case series of Chinese women with chronic iron deficiency anemia due to menorrhagia or chronic renal disease [9]. The hypothesis of oxidative stress being involved in the pathophysiology of RCVS is supported by an investigation of biomarkers for oxidative stress showing increased levels of 8-Iso-prostaglandin-F2α in RCVS patients (8-Iso-prostaglandin-F2α is a stable marker for oxidative stress originating from the peroxidation of arachidonic acid and acts as a strong vasoconstrictor) [10].

Up to now, there is limited data on the role of ferrous (Fe^2+^) and ferric (Fe^3+^) ions in vasoconstrictive syndromes: iron ions have been shown to have vasoconstrictive effects itself on the aortal system and on cerebral arteries in experimental studies [11, 12] and iron homeostasis and redox status play a key role in developing pulmonary arterial hypertension [13]. Furthermore in patients with SAH the accumulation of unbound redox-active iron has shown to be correlated with incident cerebral vasospasm [14]. Thus, iron substitution might contribute in the occurrence of cerebral vasospasms leading to RCVS.

New insights in the effect of iron in the pathophysiology of intracranial vasospasm could also be interesting for iron-binding treatment options of patients with SAH and vasospasms: in a previous study of rabbits with SAH and intracranial vasospasm the use of iron chelators reduced vasospasm by decreasing oxidative stress and endothelial cell death [15]. In primates with SAH the treatment with ferrous iron chelators prevented delayed intracranial vasospasm [16].

Furthermore, cigarette smoke exposure promotes free radical-mediated oxidative stress and is linked to endothelial dysfunction, inflammation and atherosclerosis and thus is an important cause of vascular disease [17]. Therefore, smoking might be an additional factor contributing to endothelial damage and vascular tone dysregulation in our patient.

Still, it remains unclear why the CSF opening pressure was slightly increased in our patient. The initially seen elevation of CSF opening pressure could be caused by overweight, and there have been cases reporting increased CSF pressure due to iron deficiency anemia [18, 19]. Furthermore, RCVS is known to be associated with PRES, which in turn can result in intracranial hypertension [1]. In our patient, PRES was not evident on MRI—but changes might be subtle and were possibly not present on the MRI any more as onset of the symptoms was 2 weeks prior to admission of the patient. Since the symptoms (acute severe headache) as well as the clinical and radiographic constellation were neither suggestive of intracranial hypertension nor typical MRI abnormalities reported in patients with intracranial hypertension, we saw no indication for further diagnostics and particularly treatment.

Our case might give a new perspective into the underlying pathophysiology of RCVS that is probably more frequent than previously thought. An increased understanding of the role of iron homeostasis in the regulation of cerebral vascular tone and spasms is necessary and may lead to novel insights into etiology and treatment options for RCVS. Further investigations are warranted to disentangle the complex interplay of iron homeostasis, redox balance and endothelial function in the pathophysiology of RCVS.
